# Bi-objective optimization of simultaneous vibration suppression and energy harvesting in suspension systems based on mixed H2/H∞ control

**DOI:** 10.1371/journal.pone.0358867

**Published:** 2026-09-25

**Authors:** Zhongsheng Chen, Yangyi Zhang, Ankang Wang, Guangbin Wang

**Affiliations:** 1 School of Engineering Science, Shandong Xiehe University, Jinan, Shandong, China; 2 College of Transportation and Electrical Engineering, Hunan University of Technology, Zhuzhou, Hunan, China; 3 School of Mechanical and Electrical Engineering, Lingnan Normal University, Zhanjiang, Guangdong, China; CINVESTAV IPN: Centro de Investigacion y de Estudios Avanzados del Instituto Politecnico Nacional, MEXICO

## Abstract

Simultaneous vibration suppression and energy harvesting (SVSEH) of suspension systems has attracted significant attention over the past few decades. Nevertheless, vibration suppression and energy harvesting often compete with each other due to using single-objective control methods and the tradeoff has limited the effectiveness of current systems. To reduce the tradeoff, the innovation of this paper is to propose a bi-objective control strategy in which vibration suppression and energy harvesting are optimized by two independent objective functions. Firstly, the state equation of the quarter-suspension SVSEH system is derived and nonlinear restoring force is linearized. Then the reduced-tradeoff control of the quarter-suspension SVSEH system is first equivalent to a bi-objective H2−norm optimization subject to an H∞−norm constraint. Next, the mixed H2/H∞ state feedback-based control method is presented in detail and a low-pass filter is adopted to ensure the “strictly proper” condition of H2 control. In the end, a Simulink model is built and the results indicate that it achieves a 6.2% improvement in vibration reduction and a 17.9% increase in energy harvesting compared to existing method. It confirms the proposed method can greatly reduce the tradeoff between vibration suppression and energy harvesting. Consequently, this research can contribute to developing next-generation energy-regenerative suspensions.

## Introduction

Unwanted low-frequency vibrations caused by road surface’s roughness always reduce the ride comfort and induce harmful wear on mechanical components of vehicles [1]. Therefore, suspensions are widely utilized for suppressing vibration [2]. In traditional suspension systems, springs and dampers are designed to convert vibration energy into wasted heat for dissipation. Existing data show that more than 80% of the total energy in fuel vehicles is dissipated through friction, suspension and heat emissions [3], and about 20% of the total energy in electric vehicles is dissipated by suspension systems [4]. Rather than dissipating energy through viscous damping, vibration suppression can also be achieved simultaneously if suspension systems are able to convert vibration energy into electrical energy. The history of regenerative shock absorber (RSA) can date back to 1970s, when the regeneration feasibility of suspension vibration energy was discussed [5]. Until 2010, however, RSAs began to be widely studied for suspension systems. Nowadays, simultaneous vibration suppression and energy harvesting (SVSEH) has become a hot-spot direction in the field of vehicle suspensions [6,7].

Up to now, RSAs have been widely investigated in academia and industry. Zheng et al. [8] reviewed the potential of vehicle vibration energy recovery techniques and summarized general classifications of RSAs. According to the literature, electromagnetic energy conversion mechanism is more suitable for suspension systems than both electrostatic and piezoelectric mechanism due to the harsh operation environment. Existing electromagnetic RSAs can be classified into linear-motion and rotational RSAs. As for linear-motion RSAs, the current in the coil is created by relative linear motion between the magnets and the coils. Gupta et al. [9] proposed a linear-motion RSA with a concentric arrangement of multiple inner and outer magnet rings. Zuo et al. [10, 11] designed and tested a RSA with a linear generator and the four-phase configuration provided a more even regeneration of electricity. In order to increase the magnetic field intensity, Halbach array was introduced to design the linear-motion RSA by Zhang [3]. As for rotational RSAs, Suda et al. [12–14] proposed a ball-screw electromagnetic RSA for suspension, in which linear motions from road vibrations were converted into rotational motions by the ball-screw and nut configuration. Later, Zuo et al. [15,16] first proposed a rack-pinion electromagnetic RSA, in which linear motions were transformed into rotational motions by using a rack and a pinion. However, the structures of those RSAs are mainly linear, so they cannot effectively deal with low-frequency road excitations.

According to ISO-2631 [17], ride comfort for passengers is most sensitive to vibration excitations in the 4–8 Hz range. In order to address low-frequency vibrations, nonlinear SVSEH began to attract increasing attention in recent years. Kakou and Barry [18] attached a nonlinear electromagnetic resonant shunt tuned mass-damper-inerter to a Duffing oscillator for SVSEH. Xu et al. [19] proposed a novel dual-functional nonlinear metastructure for amplitude-robust SVSEH, which consisted of bi-stable and mono-stable cubic-hardening nonlinear electromechanical resonators. In particular, quasi-zero stiffness (QZS) is a special class of nonlinear stiffness with the high-static-low-dynamic characteristics [20]. Chen et al. [21] reviewed the progress of QZS-SVSEH and outlined main challenges. Wang et al. [22] built a QZS-SVSEH prototype and the output power was 1.51mW under the excitation with a frequency of 4.5 Hz. Fang et al. [23] proposed a QZS-based monostable SVSEH device which could simultaneously broaden the vibration suppression and energy harvesting bandwidth. Yang et al. [24] proposed a high-order QZS-SVSEH device to break the bottleneck of efficient isolation and energy harvesting under ultra-low frequency excitation. Up to now, however, QZS-SVSEH has seldom been studied for suspension systems.

As for realizing SVSEH in suspension systems, four classes of control algorithms have been studied. The first category is the maximum induced power control strategy proposed by Casavola et al. [25], which transformed the optimization of RSA parameters into a linear matrix inequality (LMI) and the results demonstrated a good balance between energy harvesting and dynamic performance. The second category is the semi-active control strategy. Shi et al. [26] developed a semi-active control strategy by integrating the Skyhook with Groundhook control algorithms. Simulation results showed that this approach could well balance energy harvesting, ride comfort and handling stability under random road excitations. The third category is the predictive control strategy. Clemen et al. [27] proposed two model predictive control strategies for energy-harvesting active suspensions and the simulation results showed that the optimal balance between energy harvesting, ride comfort and handling stability was achieved. The fourth category is the mode switch control strategy. Zheng et al. [28] proposed a two-mode switching control method for electromagnetic energy harvesting suspension systems. Simulation results showed that this strategy achieved a good balance between energy harvesting and ride comfort. Wu and Zheng [29] proposed a multi-mode switching control strategy and simulation results showed that this strategy significantly improved the ride comfort while harvesting a large amount of energy simultaneously. In above control algorithms, however, single-objective optimization methods were always adopted by weighting vibration suppression and energy harvesting. In this case, vibration suppression and energy harvesting inevitably compete with each other, so the tradeoff has seriously limited the effectiveness of current SVSEH systems [30].

In order to deal with the above-mentioned issues, the authors have designed a decoupling SVSEH (D-SVSEH) structure for suspension systems by integrating QZS [31]. Nonetheless, a suitable control method remains lacking. As a result, the motivation of this paper is to advance the previous work [31] and propose a novel bi-objective control strategy to reduce the tradeoff, in which vibration suppression and energy harvesting are optimized by two independent objectives functions. In summary, the novelties of this paper can be outlined as follows.

1) A bi-objective control strategy is first proposed for the quarter-suspension SVSEH system, where the reduced-tradeoff control is transformed to a bi-objective H2-norm optimization subject to an H∞-norm constraint.
2) The 3-DOF magneto-electromechanical coupling model of the quarter-suspension SVSEH system is derived by dynamic analysis. At the same time, nonlinear restoring force of the QZS is linearized.
3) The mixed H2/H∞ state feedback-based control method is presented, where state-space models of H2-norm optimization and H∞-norm constraint are built, respectively. In particular, a low-pass filter is proposed to ensure the “strictly proper” condition of H2 control.

The remainder of the paper is structured as follows. In Sect 2, the 3-DOF magneto-electromechanical coupling model of the quarter-suspension SVSEH system is built and the corresponding issues are addressed. Then the linearized state equation of the quarter-suspension SVSEH system is built in Sect 3. The mixed H2/H∞ state feedback-based control method is presented in Sect 4. In Sect 5, a Simulink model is built to validate the proposed method. In the end, some conclusions are marked in Sect 6.

## Problem statement

**Fig 1:**
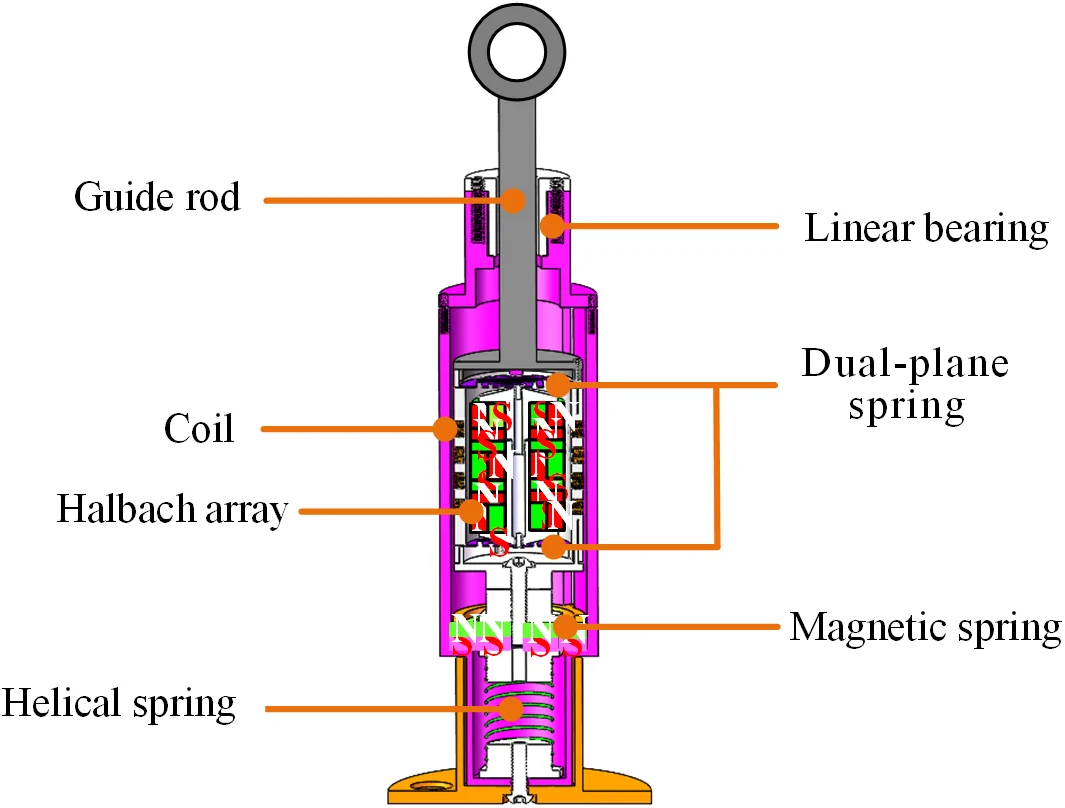
Structural diagram of the D-SVSEH structure.

The D-SVSEH structure in the previous published work [31] is shown in Fig 1. To deal with low-frequency road excitations, a pair of coaxial magnetic rings was used to generate a negative stiffness and then the QZS was formed by combing the negative stiffness and the helical spring. The electromagnetic harvester consisting of the circular Halbach array and the coil acted as the shunt damper. Particularly, the circular Halbach array was flexibly connected to the guide rod by a dual-plane spring, rather than rigid connection. The most advantage of this structure is that the payload vibration can be transferred to the electromagnetic harvester, so the conflict between vibration suppression and energy harvesting is structurally reduced.

**Fig 2:**
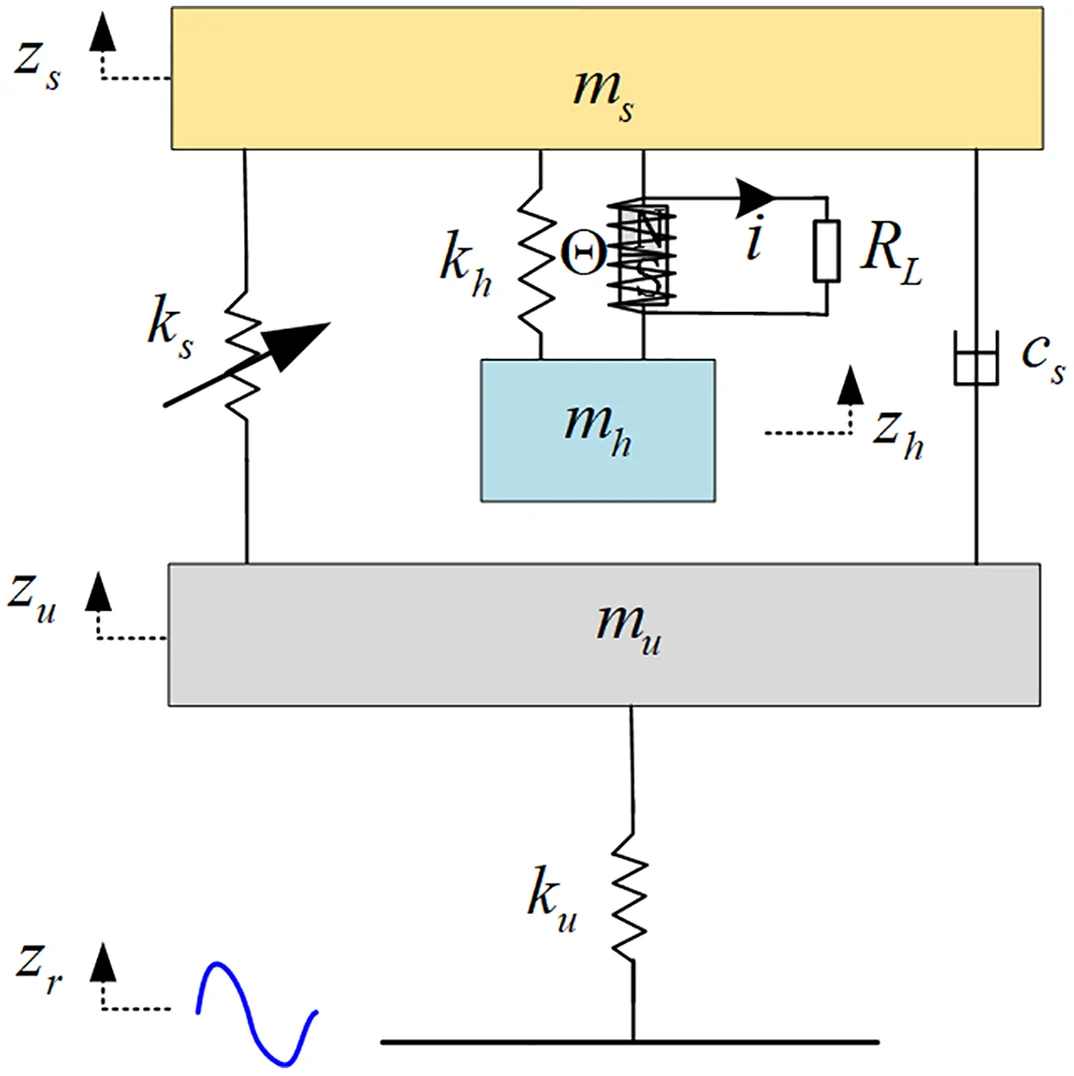
The 3-DOF quarter-suspension model including the SVSEH structure.

For the sake of simplicity, a quarter suspension including the D-SVSEH structure is considered in this paper, which can be equivalent to a 3-DOF lumped-parameter model as shown in Fig 2. In the model, $m_{h}\text{,}m_{s}\text{,}m_{u}$ are the harvester mass, the sprung mass and the unsprung mass, respectively. $z_{s}\text{,\textbackslash{}hspace\{0.33em}\}z_{u},\text{\textbackslash{}hspace\{0.33em}\}z_{h}$ are the displacements of the sprung mass, the unsprung mass and the harvester mass, respectively. $z_{r}$is the road excitation. $k_{u}$ denotes the unsprung stiffness. $k_{s}$ denotes the QZS. $k_{h}$ denotes the equivalent stiffness of the dual-plane spring. $c_{s}$ denotes the mechanical damping coefficient of the suspension system. Θdenotes the magnetoelectric coupling coefficient.iis the induced current in the coil.

According to Fig 2, the 3-DOF magneto-electromechanical coupling model of the quarter-suspension SVSEH system can be represented as follows.


$$\begin{array}{c} \left\{ \begin{array}{c} @l@m_{h}\;{\ddot{z}}_{h}=F_{h}+F_{a}\\ m_{s}\;{\ddot{z}}_{s}=-\;c_{s}(\;{\dot{z}}_{s}-\;{\dot{z}}_{u})-F_{s}-F_{h}-F_{a}\\ \;m_{u}\;{\ddot{z}}_{u}\;=c_{s}\;({\dot{z}}_{s}-{\dot{z}}_{u})\;+F_{s}-k_{u}\;(z_{u}-z_{r}) \end{array}\right. \end{array}$$ (1)

where $F_{h}\text{=}k_{h}\left(z_{s}-z_{h}\right)$ is the restoring force of the dual-plane spring, $F_{s}$ is the restoring force of the QZS spring,$F_{a}$ is the Ampere force of the current-carrying coil.

According to the Lorentz force law, $F_{a}$ can be calculated as,


$$\begin{array}{c} F_{a}=\Theta i \end{array}$$ (2)

Essentially, $F_{a}$ acts as an electromagnetic damping force.

According to road profiles, a practical energy-regenerative suspension system usually has different operation modes and their corresponding control strategies in real-world applications [32]. As for smooth cruising, the operation mode is SVSEH and the control objective to maximize energy recovery while maintaining acceptable ride comfort. While for rough roads, the operation mode is active damping. The control objective is to prioritize active vibration control and energy harvesting is paused or secondary. If necessary, the system even draws energy from the battery to supplement the damping force. This paper mainly focuses on the operation mode of SVSEH. According to energy flow under the SVSEH mode, once vibration energy of the sprung mass is harvested by the electromagnetic energy harvester, its vibration is naturally reduced. As a result, a suitable control strategy is needed to transfer vibration from the sprung mass to the harvester. Based on Eq (2), the electromagnetic damping force ($F_{a}$) can be adjusted by tuning the induced current i. In this sense, the control strategy is inherently semi-active. Different from existing systems, however, the control strategy of the quarter-suspension SVSEH system in this paper focuses on considering two independent optimization objectives, namely vibration suppression and energy harvesting. At the same time, safety requirements must also be satisfied, including the road handling stability, the suspension stroke and the maximum harvester displacement. Furthermore, the two optimization objectives and three safety constraints are described in Table 1.

**Table 1:** Description of two optimization objectives and three constraints.

Description	Property	Definition
Ride comfort	Objective	The human tolerance to vibration exposure over time
Harvested power	Objective	The average harvested power during a period
Road-handling stability	Constraint	The ability to maintain intended direction and resist skidding
Suspension stroke	Constraint	The maximum relative displacement between the unsprung and sprung mass
The maximum harvester displacement	Constraint	The maximum relative displacement of the Halbach array

Under the operation mode of SVSEH, there is no need to reduce suspension vibration to the smallest possible extent, as long as ride comfort meets the standard. That is to say, the control aim of the quarter-suspension SVSEH system is to maximize the average harvested power while satisfying the requirement of ride comfort. But there are two key challenges. On one hand, QZS brings nonlinearity into the whole system. On the other hand, two optimization objectives and three constraints are involved. To our best knowledge, existing control strategies suffer from obvious tradeoff between vibration suppression and energy harvesting, thereby limiting the harvested power. Therefore, novel control strategies need to be developed to deal with the nonlinearity and reduce the tradeoff.

## Materials and methods

State-space modeling is a powerful approach for control system design, which provides a mathematical framework for representing dynamic behaviors of mechanical systems. In particular, state-space models are much suitable for complex systems with multiple inputs and outputs, and offer a flexible way to design controllers. As a result, this idea is also introduced to develop a novel reduced-tradeoff control method of the quarter-suspension SVSEH system.

### Linearized state equation of the quarter-suspension SVSEH system

According to the previous work [33], the nonlinear restoring force of the QZS spring can be approximated as,


$$\begin{array}{c} F_{s}=k_{s\text{1}}\text{(}z_{s}-z_{u}\text{)}+k_{s\text{3}}\text{(}z_{s}-z_{u}{\text{)}}^{\text{3}} \end{array}$$ (3)

where $k_{s1}$ and $k_{s3}$ are the fitting coefficients.

This kind of nonlinearity brings a big obstacle to building the state-space model of the quarter-suspension SVSEH system. In order to overcome QZS nonlinearity, linear approximation is always adopted [34]. At the same time, QZS has an operating range around the equilibrium position, where the system exhibits near-zero dynamic stiffness. Once the displacement exceeds this critical range, it will lead to serious consequences which should be avoided in designing the QZS [35]. In this paper, the suspension stroke (i.e., $z_{s}-z_{u}$) is small, so that the QZS works within its operating range. In this case, the nonlinear restoring force can be approximately linearized. Furthermore, $F_{s}$can be rewritten as Eq (4) by introducing a small perturbation term ($F_{non}$).


$$\begin{array}{c} F_{s}={\overset{―}{k}}_{s_{\text{1}}}\text{(}z_{s}-z_{u}\text{)}+F_{non} \end{array}$$ (4)

where ${\overset{―}{k}}_{s_{\text{1}}}$ denotes the linear coefficient.

In real-world applications, the electromagnetic damping force ($F_{a}$) or the induced current (i) is tuned by a power interface circuit with a control algorithm. According to the literature [36], the input impedance of the power interface circuit can be equivalent to a pure resistance. For the sake of simplicity, this paper employs only a purely resistive load ($R_{L}$) for calculating the harvested power. In addition, the proposed bi-objective optimization aims to find the optimal ito provide adequate damping force and increase the harvested power, so the electrical dynamics of the circuit is not included in the state equations.

By substituting Eqs (2) and (4) into Eq (1), we will have


$$\begin{array}{c} \left\{ \begin{array}{c} @l@m_{h}\;{\ddot{z}}_{h}=k_{h}\text{(}z_{s}-z_{h}\text{)}+\Theta i\\ m_{s}\;{\ddot{z}}_{s}=-c_{s}\text{(}{\dot{z}}_{s}-\;{\dot{z}}_{u}\text{)}-{\overset{―}{k}}_{s\text{1}}\text{(}z_{s}-z_{u}\text{)}-k_{h}\text{(}z_{s}-z_{h}\text{)}-\Theta i-F_{non}\;\\ \;m_{u}\;{\ddot{z}}_{u}\;=c_{s}\text{(}{\dot{z}}_{s}-{\dot{z}}_{u}\text{)}+{\overset{―}{k}}_{s\text{1}}\text{(}z_{s}-z_{u}\text{)}-k_{u}\text{(}z_{u}-z_{r}\text{)+}F_{non} \end{array}\right. \end{array}$$ (5)

In order to build the state equation, six state variables are defined as follows: $x_{\text{1}}=z_{s}-z_{h}$, $x_{\text{2}}={\dot{z}}_{h}$, $x_{\text{3}}=z_{s}-z_{u}$, $x_{\text{4}}={\dot{z}}_{s}$, $x_{\text{5}}=z_{u}-z_{r}$ and $x_{\text{6}}={\dot{z}}_{u}$. At the same time, both $F_{non}$ and the road excitation are considered as perturbation terms, leading to the perturbation vector $𝐰={\left[F_{non,}\text{\textbackslash{}hspace\{0.33em}\}{\dot{z}}_{r}\right]}^{T}$. Then the linearized state equation can be represented as Eq (6) based on Eq (5).


$$\begin{array}{c} \dot{𝐱}=𝐀𝐱+{𝐁}_{u}u+{𝐁}_{w}𝐰 \end{array}$$ (6)

where $𝐱={\left[x_{\text{1}}x_{\text{2}}x_{\text{3}}x_{\text{4}}x_{\text{5}}x_{\text{6}}\right]}^{T}$is the state vector, u denotes the control input, $𝐀,\text{\textbackslash{}hspace\{0.33em}\}{𝐁}_{u}\text{,}{𝐁}_{w}$are constant matrices defined as follows.

$𝐀=\left[\begin{array}{cccccc} @cccccc@0 & -1 & 0 & 1 & 0 & 0\\ \frac{k_{h}}{m_{h}} & 0 & 0 & 0 & 0 & 0\\ 0 & 0 & 0 & 1 & 0 & -1\\ -\frac{k_{h}}{m_{s}} & 0 & -\frac{k_{s1}}{m_{s}} & -\frac{c_{s}}{m_{s}} & 0 & \frac{c_{s}}{m_{s}}\\ 0 & 0 & 0 & 0 & 0 & 1\\ 0 & 0 & \frac{k_{s1}}{m_{u}} & \frac{c_{s}}{m_{u}} & -\frac{k_{u}}{m_{u}} & -\frac{c_{s}}{m_{u}} \end{array}\right]$, $B_{\text{u}}=\left[\begin{array}{c} @c@0\\ \mathrm{\backslash raisebox}1ex\backslash (\Theta \backslash )\;/\;\mathrm{\backslash raisebox}\mathrm{-1ex}\backslash (m_{h}\backslash )\\ 0\\ -\mathrm{\backslash raisebox}1ex\backslash (\Theta \backslash )\;/\;\mathrm{\backslash raisebox}\mathrm{-1ex}\backslash (m_{s}\backslash )\\ 0\\ 0 \end{array}\right]$, ${𝐁}_{w}=\left[\begin{array}{cc} @cc@0 & 0\\ 0 & 0\\ 0 & 0\\ -\mathrm{\backslash raisebox}1ex\backslash (1\backslash )\;/\;\mathrm{\backslash raisebox}\mathrm{-1ex}\backslash (m_{s}\backslash ) & 0\\ 0 & -1\\ \mathrm{\backslash raisebox}1ex\backslash (1\backslash )\;/\;\mathrm{\backslash raisebox}\mathrm{-1ex}\backslash (m_{u}\backslash ) & 0 \end{array}\right]$.

Next, in order to formulate the control strategy of the quarter-suspension SVSEH system, the two optimization objectives and three constraints listed in Table 1 need to be defined based on the state variables.

*Ride comfort* is often based on measurements of vibration accelerations, which can be quantified by a ride comfort index. In ISO2631 standard, the ride comfort index is usually defined as the root mean square (RMS) of x-, y- and z-axis accelerations. In this paper, however, only the vertical acceleration of the sprung mass is considered due to focusing on vertical vibration. Then the optimization objective of ride comfort is defined as,


$$\begin{array}{c} min{\ddot{z}}_{s}\left(t\right) \end{array}$$ (7)

*Harvested power* refers to the average power consumed on the resistive load ($R_{L}$) during a given period T. Then the optimization objective of the harvested power is defined as,


$$\begin{array}{c} maxP_{h}\text{=}max\left\{\frac{\text{1}}{T}\int_{\text{0}}^{T}i^{\text{2}}\left(t\right)R_{L}dt\right\} \end{array}$$ (8)

*The road-handling stability* is usually represented by the tyre deflection. Then the constraint of the road-handling stability is defined as,


$$\begin{array}{c} \frac{k_{u}(z_{u}-z_{r})}{(m_{s}+m_{u}+m_{h})g}<1 \end{array}$$ (9)

*The suspension stroke* refers to the maximum relative vertical displacement ($z_{max}$) of the suspension. Then the constraint of the suspension vibration is defined as,


$$\begin{array}{c} \left|z_{s}-z_{u}\right|<z_{max} \end{array}$$ (10)

*The maximum harvester displacement* refers to the maximum relative vertical displacement ($z_{hmax}$) of the Halbach array. Then the constraint of the harvester vibration is defined as,


$$\begin{array}{c} \left|z_{h}-z_{s}\right|<z_{hmax} \end{array}$$ (11)

### Mixed H2/H∞ state feedback-based control method

Nowadays LMI provides a powerful tool for dealing with multiple objectives in control problems [37]. In particular, LMI is fit for designing controllers that simultaneously satisfy multiple metrics, such as H2 [38] and H∞ controllers [39]. According to the literature [40], H2/H∞ synthesis is much fit for optimizing a controller while considering several objectives/constraints. As a result, H2/H∞ synthesis can provide a promising solution to the above quarter-suspension SVSEH system, which has rarely been studied before.

**Fig 3:**
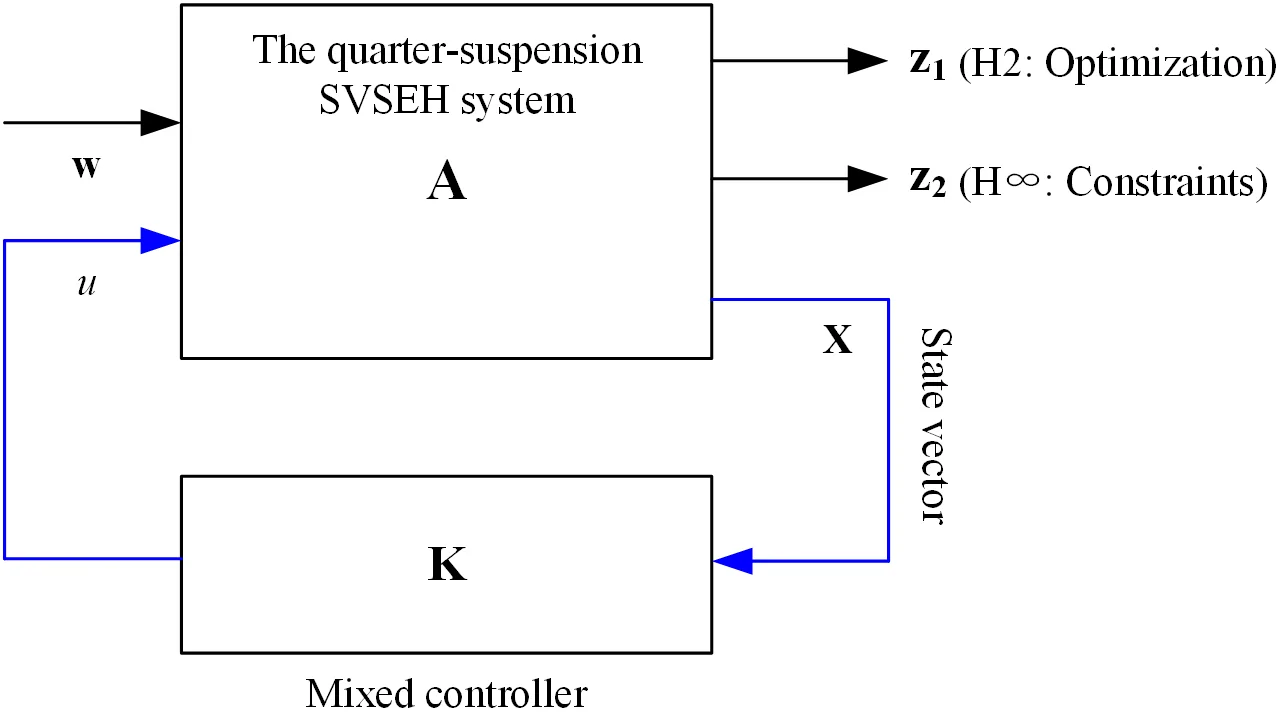
The framework of the mixed H2/H∞ state feedback-based control strategy.


In this paper, the framework of mixed H2/H∞ state feedback-based control strategy is presented as Fig 3. The quarter-suspension SVSEH system is represented by the real-rational matrix 𝐀 which is defined by the linearized model. 𝐊is the state feedback gain matrix. uis the control input. 𝐱is the state vector. ${𝐳}_{\text{1}},\text{\textbackslash{}hspace\{0.33em}\}{𝐳}_{\text{2}}$ denote the optimization objective vector and the constraint vector, respectively. Furthermore, ${𝐓}_{\text{1}}\left(𝐊\right)\text{,\textbackslash{}hspace\{0.33em}\}{𝐓}_{\text{2}}\left(𝐊\right)$ denote the closed-loop transfer matrices from 𝐰to ${𝐳}_{\text{1}}$and ${𝐳}_{\text{2}}$, respectively. Then the mixed H2/H∞ controller is refined as an H2-norm optimization subject to an H∞-norm constraint. That is to say,

$$\begin{array}{c} \left\{𝐊{|\text{min}\|{𝐓}_{\text{1}}\left(𝐊\right)\|}_{\text{2}}\text{\textbackslash{}hspace\{0.33em}and\;\;\}{\|{𝐓}_{\text{2}}\left(𝐊\right)\|}_{\infty }<\gamma \right\} \end{array}$$ (12)

where γ is a small positive scalar indicating the tradeoff factor. Next, the challenging task is to solve the control law.

In theory, the mixed H2/H∞ framework is fundamentally about tradeoff control, which provides a systematic way to balance two competing control objectives: H_2_ optimal performances and H∞ constraints. In this paper, on the one hand, ride comfort and harvested power are two separate objective functions defined in the H_2_ norm optimization, so the tradeoff between them is greatly reduced. On the other hand, the H∞ constraint is closely related to driving safety, so it must be satisfied. At the same time, there exists a tradeoff factor (γ) that is used to reconcile the conflict between the H_2_ optimization and the H∞ constraint, which also affects ride comfort and harvested power. Therefore, the above mixed H2/H∞ framework for the quarter-suspension SVSEH system is referred to as a reduced‑tradeoff control strategy.

#### A. State-space model of the H2 control.

As shown in Fig 3, ${𝐳}_{\text{1}}$is defined based on the ride comfort and the harvested power. Obviously, the transfer function ($H_{{\dot{z}}_{r}\to {\ddot{z}}_{s}}$) from ${\dot{z}}_{r}$to ${\ddot{z}}_{s}$ definitely exists, so ${\ddot{z}}_{s}$ can be used as a component of ${𝐳}_{\text{1}}$. Based on Eq (6), the harvested power depends on the mean square of the induced current, which is defined as $i_{RMS}^{\text{2}}=\frac{\text{1}}{T}\int_{\text{0}}^{T}i{\left(t\right)}^{\text{2}}dt$. Then it needs to judge whether $i_{RMS}^{\text{2}}$can be represented by the transfer function ($H_{{\dot{z}}_{r}\to i}$) from ${\dot{z}}_{r}$to i or not.

According to the Parseval theorem, $i_{RMS}^{\text{2}}$ can be rewritten as follows in the frequency domain.


$$\begin{array}{c} i_{RMS}^{\text{2}}=\frac{\text{1}}{T}\int_{\text{0}}^{T}i{\left(t\right)}^{\text{2}}dt=\frac{\text{1}}{2\pi }\int_{-\infty }^{+\infty }{\left|H_{{\dot{z}}_{r}\to i}\left(j\omega \right)\right|}^{\text{2}}S_{{\dot{z}}_{r}}\left(\omega \right)d\omega \end{array}$$ (13)

where$S_{{\dot{z}}_{r}}\left(\omega \right)$ is the power spectral density (PSD) of the road velocity excitation. At the same time, we will have,


$$\begin{array}{c} {\|H_{{\dot{z}}_{r}\to i}\|}_{\text{2}}^{\text{2}}=\frac{\text{1}}{2\pi }\int_{-\infty }^{+\infty }{\left|H_{{\dot{z}}_{r}\to i}\left(j\omega \right)\right|}^{\text{2}}d\omega \end{array}$$ (14)

According to ISO 8608 [41], the spatial PSD of road velocity excitation can be written as follows.


$$\begin{array}{c} G_{z_{r}}\left(n\right)=G_{z_{r}}\left(n_{\text{0}}\right){\left(n/n_{\text{0}}\right)}^{-\text{2}} \end{array}$$ (15)

where, $n_{\text{0}}\text{=0.1\textbackslash{}hspace\{0.33em}rad/m\}$, $G_{z_{r}}\left(n_{\text{0}}\right)$is the baseline of spatial PSD at $n_{\text{0}}\text{=0.1\textbackslash{}hspace\{0.33em}rad/m\}$, and n denotes the spatial frequency.

On the other hand, the temporal frequency can be calculated as f=nv, where v is the vehicle velocity. Then the temporal PSD of road velocity excitation can be derived as follows based on Eq (15).


$$\begin{array}{c} S_{z_{r}}\left(f\right)=G_{z_{r}}\left(n_{\text{0}}\right)\frac{v}{f^{\text{2}}} \end{array}$$ (16)

Then the temporal PSD of road velocity excitation can be calculated as,


$$\begin{array}{c} S_{{\dot{z}}_{r}}\left(f\right)={\left|j\text{2}\pi f\right|}^{\text{2}}S_{z_{r}}\left(f\right)=4\pi^{\text{2}}vG_{z_{r}}\left(n_{\text{0}}\right) \end{array}$$ (17)

It can be seen from Eq (17) that $S_{{\dot{z}}_{r}}\left(f\right)$ is independent of both f and ω. By combining Eqs (13), (14) and (17), we will have,


$$\begin{array}{c} i_{RMS}^{\text{2}}=4\pi^{\text{2}}vG_{z_{r}}\left(n_{\text{0}}\right){\|H_{{\dot{z}}_{r}\to i}\|}_{\text{2}}^{\text{2}} \end{array}$$ (18)

Obviously, Eq (18) shows that $i_{RMS}^{\text{2}}$ is proportional to ${\|H_{{\dot{z}}_{r}\to i}\|}_{\text{2}}^{\text{2}}$. Therefore, it is proved that the harvested power can also be optimized by the *H*_2_ norm. In order to facilitate the minimization operation, −i is selected as the other component of ${𝐳}_{\text{1}}$. Then the optimization objective vector is defined as,


$$\begin{array}{c} {𝐳}_{1}={\left[{\ddot{z}}_{s}-i\right]}^{T} \end{array}$$ (19)

Thus the state-space model of the H2 control can be written as,


$$\begin{array}{c} \left\{ \begin{array}{c} @l@\dot{𝐱}=𝐀𝐱+{𝐁}_{u}u+{𝐁}_{w}𝐰\\ {𝐳}_{\text{1}}={𝐂}_{\text{1}}𝐱+{𝐂}_{1u}u+{𝐂}_{1w}𝐰 \end{array}\right. \end{array}$$ (20)

where ${𝐂}_{\text{1}}\text{,\textbackslash{}hspace\{0.33em}\;\}{𝐂}_{\text{1}u}\text{,\textbackslash{}hspace\{0.33em}\}{𝐂}_{\text{1}w}$ are constant matrices defined as follows.


$$\begin{array}{c} {𝐂}_{\text{1}}=\left[\begin{array}{cccccc} -k_{h}/m_{s} & 0 & -k_{s1}/m_{s} & -c_{s}/m_{s} & 0 & c_{s}/m_{s}\\ 0 & 0 & 0 & 0 & 0 & 0 \end{array}\right],\text{\textasciitilde{}}{𝐂}_{\text{1}u}=\left[\begin{array}{c} -\Theta /m_{s}\\ -\text{1} \end{array}\right],\text{\textasciitilde{}}{𝐂}_{1w}=\left[\begin{array}{cc} -\text{1}/m_{s} & \text{0}\\ \text{0} & \text{0} \end{array}\right]\text{.} \end{array}$$

According to the H2 control theory, the H2 norm does not necessarily exist if the transfer function is not strictly proper. Unfortunately, ${𝐂}_{1w}$ is a non-zero matrix, so it is difficult to ensure the “strictly proper” condition in the whole frequency domain due to the high-order nonlinear term ($F_{non}$) included in 𝐰. To overcome this issue, a first-order low-pass filter is defined to suppress high-frequency interrupts due to $F_{non}$, which is represented as,


$$\begin{array}{c} \alpha \left(s\right)=\frac{\omega_{\text{0}}}{s+\omega_{\text{0}}} \end{array}$$ (21)

where $\omega_{\text{0}}=2\pi f_{\text{0}}$ and $f_{\text{0}}$ is the cut-off frequency of the low-pass filter. By this way, the “strictly proper” condition can be definitely satisfied.

Then a new state variable ($x_{\text{7}}$) is defined as $X_{\text{7}}\left(s\right)=\alpha \left(s\right)Z_{\text{1}1}\left(s\right)$ in the Laplace domain. Based on it, the time-domain differential equation can be obtained as,


$$\begin{array}{c} {\dot{x}}_{\text{7}}=-\omega_{\text{0}}x_{\text{7}}+\omega_{\text{0}}z_{\text{1}1} \end{array}$$ (22)

where $z_{11}={\ddot{z}}_{s}$, $Z_{\text{11}}\left(s\right),\text{\textbackslash{}hspace\{0.33em}\}X_{\text{7}}\left(s\right)$are the Laplace transform of $z_{11}$ and $x_{\text{7}}$, respectively.

Then Eq (20) can be rewritten as an augmented state-space model as follows,


$$\begin{array}{c} \left\{ \begin{array}{c} @l@{\dot{𝐱}}_{a}={𝐀}_{a}{𝐱}_{a}+{𝐁}_{au}u+{𝐁}_{aw}𝐰\\ {𝐳}_{1a}={𝐂}_{1a}{𝐱}_{a}+{𝐂}_{1au}u+{𝐂}_{1aw}𝐰 \end{array}\right. \end{array}$$ (23)

where ${𝐱}_{a}={\left[x_{\text{1}}x_{\text{2}}x_{\text{3}}x_{\text{4}}x_{\text{5}}x_{\text{6}}\text{\textbackslash{}hspace\{0.33em}\;\}x_{\text{7}}\right]}^{T}$ and ${𝐀}_{a},\text{\textbackslash{}hspace\{0.33em}\}{𝐁}_{au}\text{,}{𝐁}_{aw}$, ${𝐂}_{\text{1a}}\text{,\textbackslash{}hspace\{0.33em}\;\}{𝐂}_{\text{1a}u}\text{,\textbackslash{}hspace\{0.33em}\}{𝐂}_{\text{1a}w}$ are defined as follows.


$$\begin{array}{c} {𝐀}_{a}=\left[\begin{array}{ccccccc} 0 & -1 & 0 & 1 & 0 & 0 & 0\\ k_{h}/m_{h} & 0 & 0 & 0 & 0 & 0 & 0\\ 0 & 0 & 0 & 1 & 0 & -1 & 0\\ -k_{h}/m_{s} & 0 & -k_{s1}/m_{s} & -c_{s}/m_{s} & 0 & c_{s}/m_{s} & 0\\ 0 & 0 & 0 & 0 & 0 & 1 & 0\\ 0 & 0 & k_{s1}/m_{u} & c_{s}/m_{u} & -k_{u}/m_{u} & -c_{s}/m_{u} & 0\\ -\omega_{\text{0}}k_{h}/m_{s} & 0 & -\omega_{\text{0}}k_{s1}/m_{s} & -\omega_{\text{0}}c_{s}/m_{s} & 0 & \omega_{\text{0}}c_{s}/m_{s} & -\omega_{\text{0}} \end{array}\right],\text{\textasciitilde{}}{𝐁}_{\text{au}}=\left[\begin{array}{c} 0\\ \Theta /m_{h}\\ 0\\ -\Theta /m_{s}\\ 0\\ 0\\ -\omega_{\text{0}}\Theta /m_{s} \end{array}\right]\text{.} \end{array}$$

$$\begin{array}{c} {𝐁}_{aw}={\left[\begin{array}{ccccccc} @ccccccc@\text{0} & \text{0} & \text{0} & -\text{1}/m_{s} & \text{0} & \text{1}/m_{u} & -\omega_{\text{0}}/m_{s}\\ \text{0} & \text{0} & \text{0} & \text{0} & -\text{1} & \text{0} & \text{0} \end{array}\right]}^{T}\text{,\textasciitilde{}}{𝐂}_{1a}=\left[\begin{array}{ccccccc} \text{0} & \text{0} & \text{0} & \text{0} & \text{0} & \text{0} & \text{1}\\ \text{0} & \text{0} & \text{0} & \text{0} & \text{0} & \text{0} & \text{0} \end{array}\right],\text{\textasciitilde{}}{𝐂}_{\text{1a}u}=\left[\begin{array}{c} \text{0}\\ -\text{1} \end{array}\right],{𝐂}_{1aw}=0_{\text{2}\times \text{2}}\text{.} \end{array}$$

#### B. State-space model of the H∞ control.

The proposed controller must also meet the three constraints, which can be ensured by the constraint of $\text{\textbackslash{}hspace\{0.33em}\}{\|{𝐓}_{\text{2}}\left(𝐊\right)\|}_{\infty }<\gamma$. Based on the definitions in Eqs (9)–(11), the constraint vector ${𝐳}_{\text{2}}$ can be denoted as,


$$\begin{array}{c} {𝐳}_{\text{2}}={\left[\frac{z_{s}-z_{u}}{z_{max}}\;\frac{z_{h}-z_{s}}{z_{hmax}}\;\frac{k_{u}(z_{u}-z_{r})}{(m_{h}+m_{s}+m_{u})g}\right]}^{T} \end{array}$$ (24)

Then the state-space model of the H∞ control can be written as,


$$\begin{array}{c} \left\{ \begin{array}{c} @l@{\dot{𝐱}}_{a}={𝐀}_{a}{𝐱}_{a}+{𝐁}_{au}u+{𝐁}_{aw}𝐰\\ {𝐳}_{\text{2}}={𝐂}_{\text{2}}{𝐱}_{a}+{𝐂}_{2u}u+{𝐂}_{2w}𝐰 \end{array}\right. \end{array}$$ (25)

where ${𝐂}_{\text{2}}\text{,\textbackslash{}hspace\{0.33em}\;\}{𝐂}_{\text{2}u}\text{,\textbackslash{}hspace\{0.33em}\}{𝐂}_{\text{2}w}$ are constant matrices defined as follows.


$$\begin{array}{c} {𝐂}_{\text{2}}=\left[\begin{array}{ccccccc} 0 & 0 & 1/z_{max} & 0 & 0 & 0 & 0\\ 1/z_{hmax} & 0 & 0 & 0 & 0 & 0 & 0\\ 0 & 0 & 0 & 0 & k_{u}/(m_{h}+m_{s}+m_{u})g & 0 & 0 \end{array}\right],\text{\textasciitilde{}}{𝐂}_{2u}=0_{3\times 1},\text{\textasciitilde{}}{𝐂}_{2w}=0_{3\times \text{2}}\text{.} \end{array}$$

#### C. LMIs-based solution of the mixed H2/H∞ controller.

For the above reduced-tradeoff control problem, the state-feedback control law is defined as Eq (26)*.*


$$\begin{array}{c} u=𝐊{𝐱}_{a} \end{array}$$ (26)

Then the aim is to derive the proper solution of 𝐊 based on LMIs.

Firstly, the state-space model corresponding to the *H*_2_ control are rewritten as,


$$\begin{array}{c} \left\{ \begin{array}{c} @l@{\dot{𝐱}}_{a}=\left({𝐀}_{a}+{𝐁}_{au}𝐊\right){𝐱}_{a}+{𝐁}_{aw}𝐰\\ {𝐳}_{\text{1a}}=\left({𝐂}_{\text{1a}}+{𝐂}_{\text{1a}u}𝐊\right){𝐱}_{a}+{𝐂}_{\text{1a}w}𝐰 \end{array}\right. \end{array}$$ (27)

As for Eq (27), the LMI formulation for the H2 control can be represented as,


$$\begin{array}{c} \left(\begin{array}{cc} @cc@𝐙 & {𝐂}_{\text{1a}}𝐗+{𝐂}_{\text{1a}u}𝐘\\ {}* & 𝐗 \end{array}\right)>0 \end{array}$$ (28)

where 𝐗,\hspace{0.33em}𝐘,\hspace{0.33em}𝐙 are positive definite matrices to be solved.

Secondly, the state-space model corresponding to the H∞ control are rewritten as,


$$\begin{array}{c} \left\{ \begin{array}{c} @l@{\dot{𝐱}}_{a}=\left({𝐀}_{a}+{𝐁}_{au}𝐊\right){𝐱}_{a}+{𝐁}_{aw}𝐰\\ {𝐳}_{\text{2}}=\left({𝐂}_{\text{2}}+{𝐂}_{\text{2}u}𝐊\right){𝐱}_{a}+{𝐂}_{\text{2}w}𝐰 \end{array}\right. \end{array}$$ (29)

Then the LMI formulation for the H∞ control can be represented as Eq (30).


$$\begin{array}{c} \left(\begin{array}{ccc} @ccc@{\left({𝐀}_{a}𝐗+{𝐁}_{au}𝐘\right)}^{\text{T}}+{𝐀}_{a}𝐗+{𝐁}_{au}𝐘 & * & *\\ {𝐁}_{aw}^{T} & -\gamma 𝐈 & *\\ {𝐂}_{\text{2}}𝐗+{𝐂}_{\text{2}u}𝐘 & {𝐂}_{\text{2}w} & -\gamma 𝐈 \end{array}\right)<0 \end{array}$$ (30)

Similarly, 𝐗,\hspace{0.33em}𝐘,\hspace{0.33em}𝐙 in Eq (30) can be analytically solved.

In the end, the common solution (${𝐗}_{c},\text{\textbackslash{}hspace\{0.33em}\}{𝐘}_{c},\text{\textbackslash{}hspace\{0.33em}\}{𝐙}_{c}$) of both Eq (28) and Eq (30) can be obtained. Then the feedback gain matrix of the mixed H2/H∞ controller can be calculated as


$$\begin{array}{c} {𝐊}_{mix}={𝐘}_{c}{𝐗}_{c}^{-1} \end{array}$$ (31)

## Case study

### Matlab/Simulink model of the quarter-suspension SVSEH system

**Fig 4:**
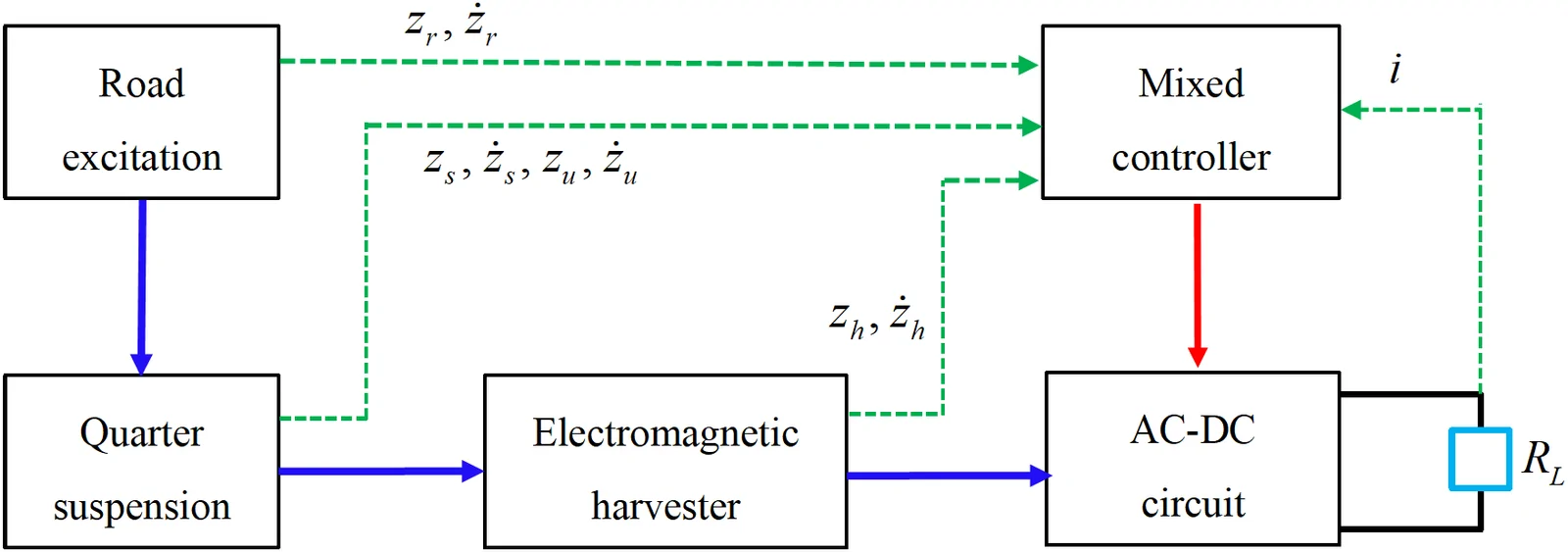
The whole framework of the Matlab/Simulink simulation model.

**Fig 5:**
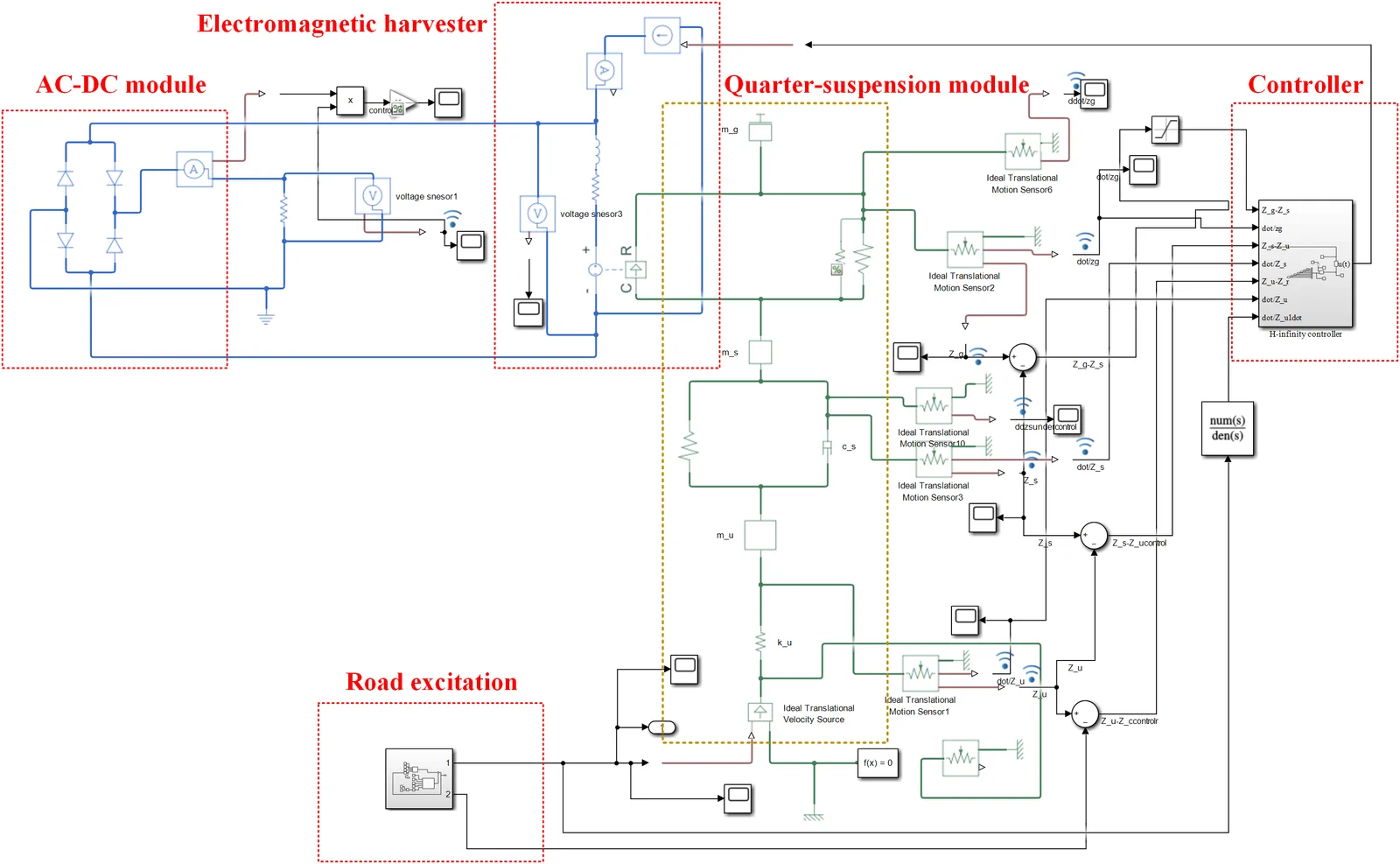
Simulink model of the quarter-suspension SVSEH system.

In order to validate the effectiveness of the proposed mixed H2/H∞ control strategy, a Matlab/Simulink model of the quarter-suspension SVSEH system is built here. Firstly, the whole framework is shown in Fig 4, which consists of five modules including the road excitation module, the quarter-suspension module, the electromagnetic harvester module, the mixed controller module and the AC-DC module. Then each module is carried out by using the corresponding Simulink blocks and the whole Simulink model is shown in Fig 5.

### Simulation of the road excitation

According to ISO 8608, andom road profiles can be classified into eight categories A ~ H based on the spatial PSD. Furthermore, time-domain formula of each road profile can be represented by the following first-order differential equation [42].


$$\begin{array}{c} {\dot{z}}_{r}\left(t\right)=-2\pi n_{c}z_{r}\left(t\right)+2\pi n_{\text{0}}\sqrt{G_{z_{r}}\left(n_{\text{0}}\right)v}\zeta \left(t\right) \end{array}$$ (32)

where $n_{c}\text{=0.01}\times v$ is the spatial cutoff frequency, ζ(t) is the road input which is a filtered white noise, and $G_{z_{r}}\left(n_{\text{0}}\right)$is related to the class of road profile. As for Class C roads, $G_{z_{r}}\left(n_{\text{0}}\right)\text{=1.6}\times {\text{10}}^{-5}$.

**Fig 6:**
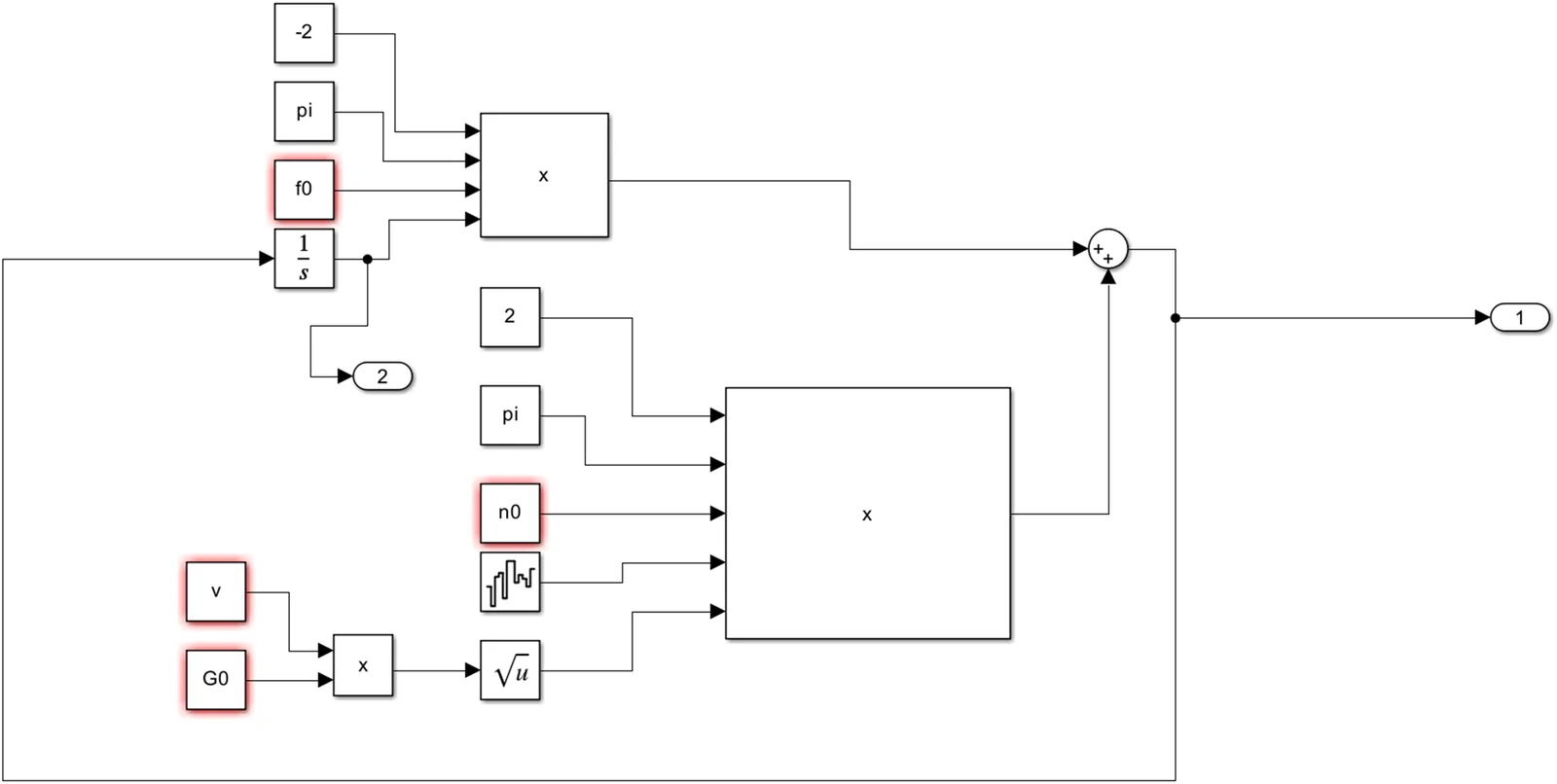
Simulink-based road excitation module.

Based on Eq (32), the road excitation module is built in the Matlab/Simulink, as shown in Fig 6.

### Results

Simulation parameter values of the quarter-suspension SVSEH system are listed in Table 2. Based on the above Simulink model, dynamic behaviors of both the quarter suspension and the electromagnetic harvester can be simulated and analyzed.

**Table 2:** Parameter values in the simulations.

Description	Symbol	Value	Unit
**Sprung mass**	$m_{s}$	200	Kg
**Unsprung mass**	$m_{u}$	20	Kg
**Tire stiffness**	$k_{u}$	320000	$\text{N}\cdot {\text{m}}^{-\text{1}}$
**Mechanical damping**	$c_{s}$	700	$\text{N}\cdot \text{s}\cdot {\text{m}}^{-\text{1}}$
**The suspension stroke**	$z_{max}$	0.02	m
**The harvester mass**	$m_{h}$	2	Kg
**The maximum harvester displacement**	$z_{hmax}$	0.05	m
**Linear part of the QZS**	${\overset{―}{k}}_{s1}$	−16141	$\text{N}\cdot {\text{m}}^{\text{-1}}$
**Stiffness of the dual-plane spring**	$k_{h}$	20000	$\text{N}\cdot {\text{m}}^{\text{-1}}$
**Resistive load**	$R_{L}$	2000	Ω
**Total inductance of the coil**	$L_{coil}$	5	mH
**Cut-off frequency**	$f_{\text{0}}$	100	Hz

**Fig 7:**
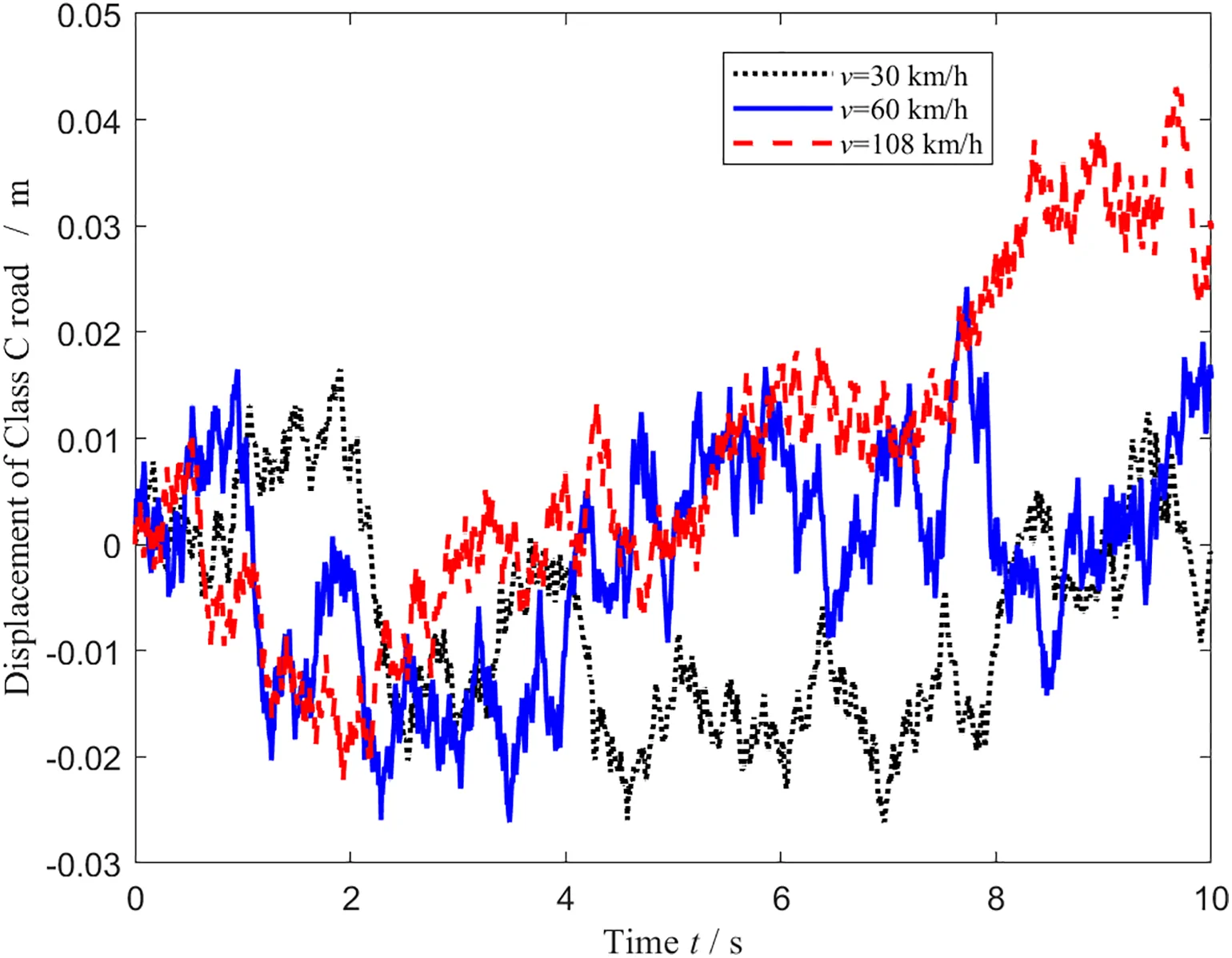
Class C road profiles under different v.

To simulate non-rough road surfaces, vibration excitations of Class C roads under three vehicle velocities (30 km/h, 60 km/h and 108 km/h) are simulated and plotted in Fig 7, respectively. It can be seen that the road excitation amplitude increases with the vehicle velocity. Furthermore, the Class C road profile at v=108km/h is adopted as the road excitation of subsequent simulations.

Next, the feedback gain matrix of the mixed H2/H∞ controller is calculated as,


$$\begin{array}{c} {𝐊}_{mix}=\left[-56.4241\;-0.8393\text{0}\mathrm{.9304}\text{0.6238 206.1124}-0\text{.0259 0.6400}\right] \end{array}$$ (33)

**Fig 8:**
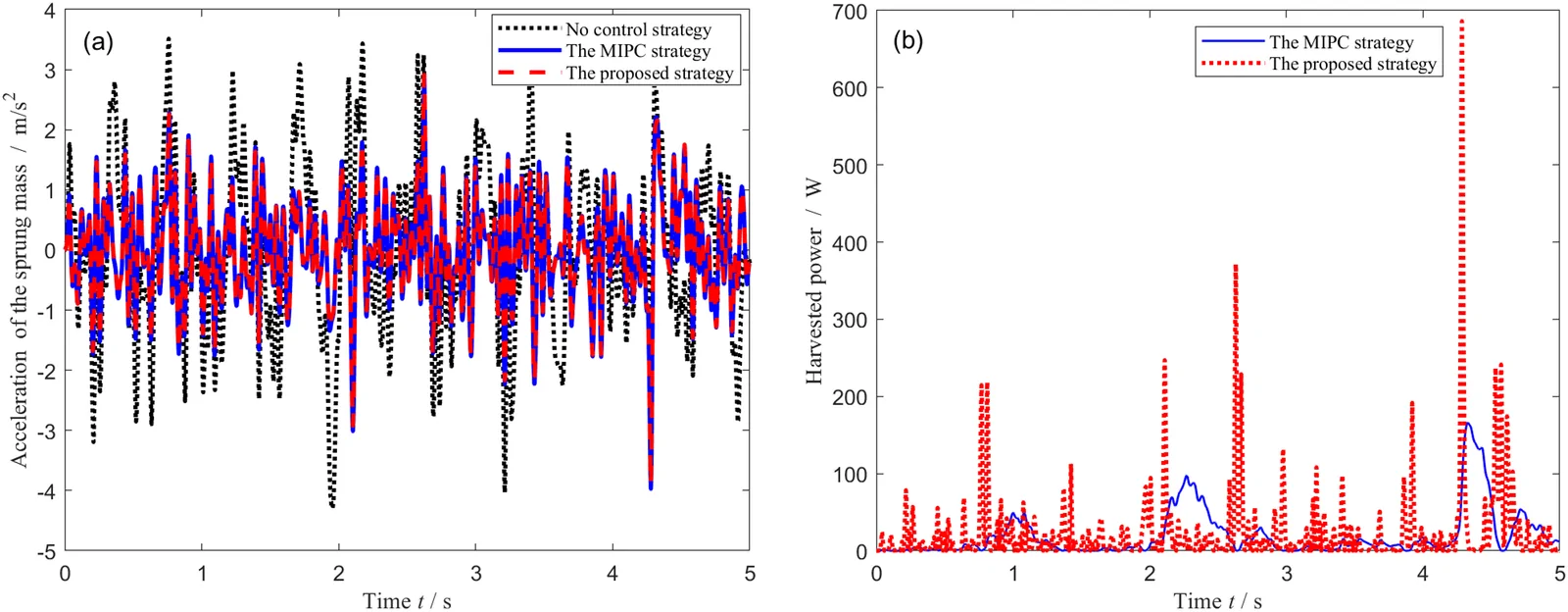
Comparison of (a) vibration suppression and (b) energy harvesting under Class C road excitation.

**Fig 9:**
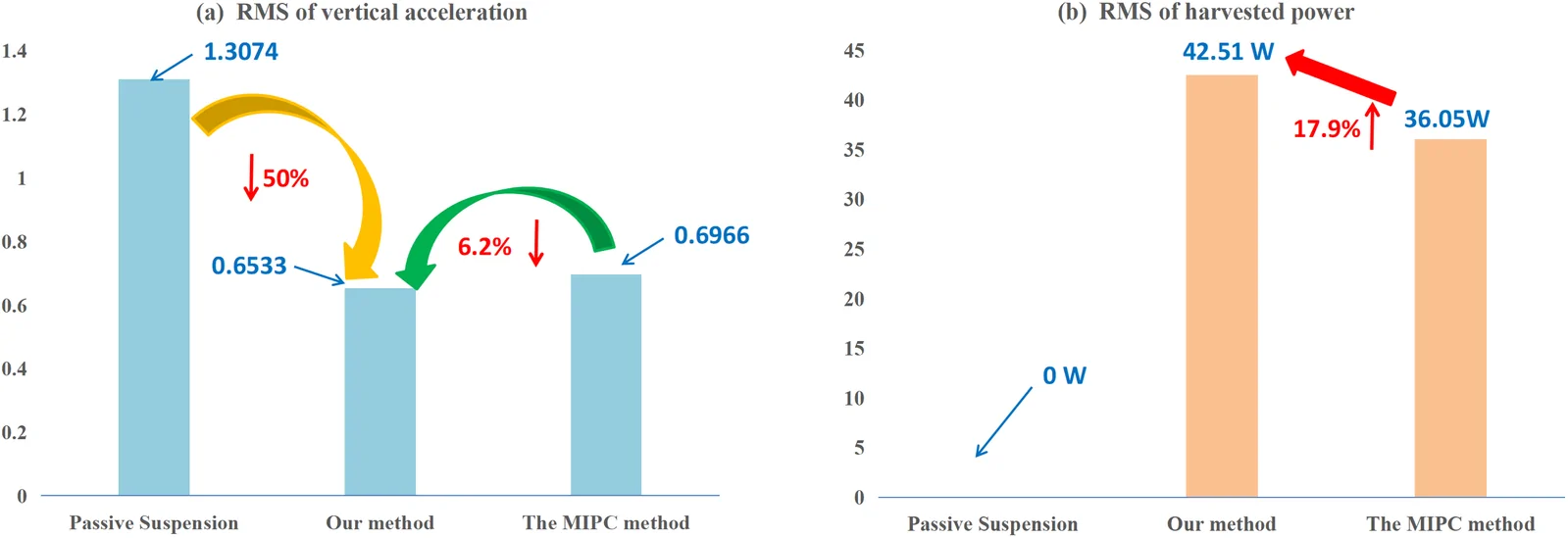
RMSs of the (a) vertical acceleration and (b) harvested power for three cases.

Finally, other state-of-the-art control strategies of SVSEH systems are also investigated here in order to show the superiority of the proposed control strategy. To our best knowledge, the maximum induced power control (MIPC) strategy proposed by Casavola et al. [25] was the only control strategy in which energy harvesting was looked as an independent objective in vehicle suspension systems. Therefore, the MIPC strategy is used as the benchmark in this paper. Under the same Class C road excitation, vertical accelerations and the harvested powers under two different control strategies and the passive case are simulated. All results are plotted in Fig 8 and the RMS values are compared in Fig 9, respectively.

## Discussion

### Comparison with state-of-the-art control strategies

In the above simulations, two different control strategies and the passive case are considered, including the proposed control strategy (i.e., mixed H2/H∞), the MIPC strategy and passive suspension. It can be seen from Fig 9 that: i) Compared with passive suspension, semi-active suspensions (including the proposed and the MIPC strategies) can more effectively suppress vertical vibrations of the sprung mass. As a result, effective semi-active control strategies can greatly improve the ride comfort; ii) RMS of vertical acceleration of the proposed method is 50% less than that of passive suspension and 6.2% less than that of the MIPC strategy; iii) Compared with the MIPC strategy, the proposed method can collect 17.9% more electric energy. These results demonstrate that the mixed H2/H∞ control strategy performs better than the MIPC strategy. That is to say, the tradeoff between vibration suppression and energy harvesting is reduced by using the proposed control strategy.

**Fig 10:**
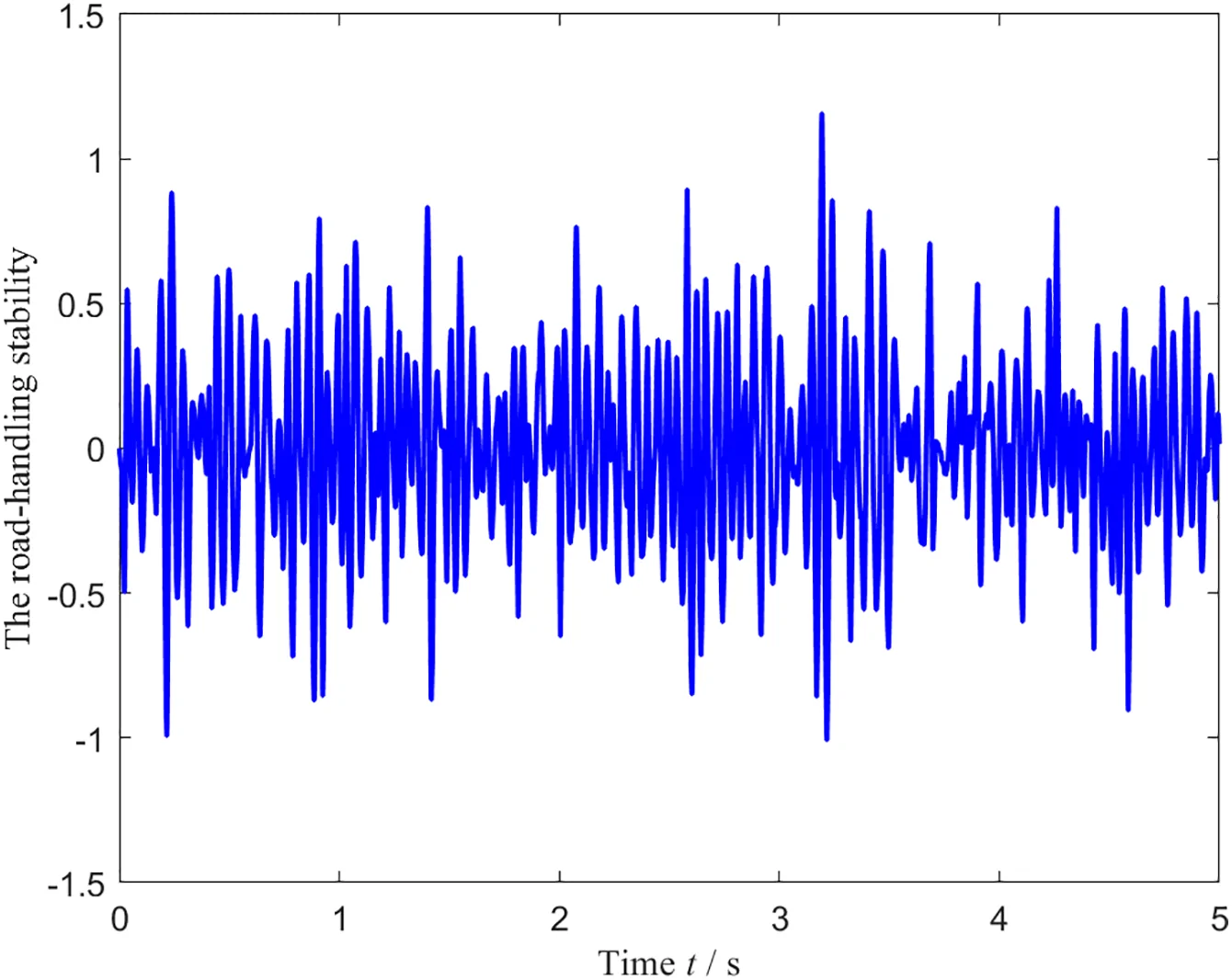
The road-handling stability under the Class C road.

**Fig 11:**
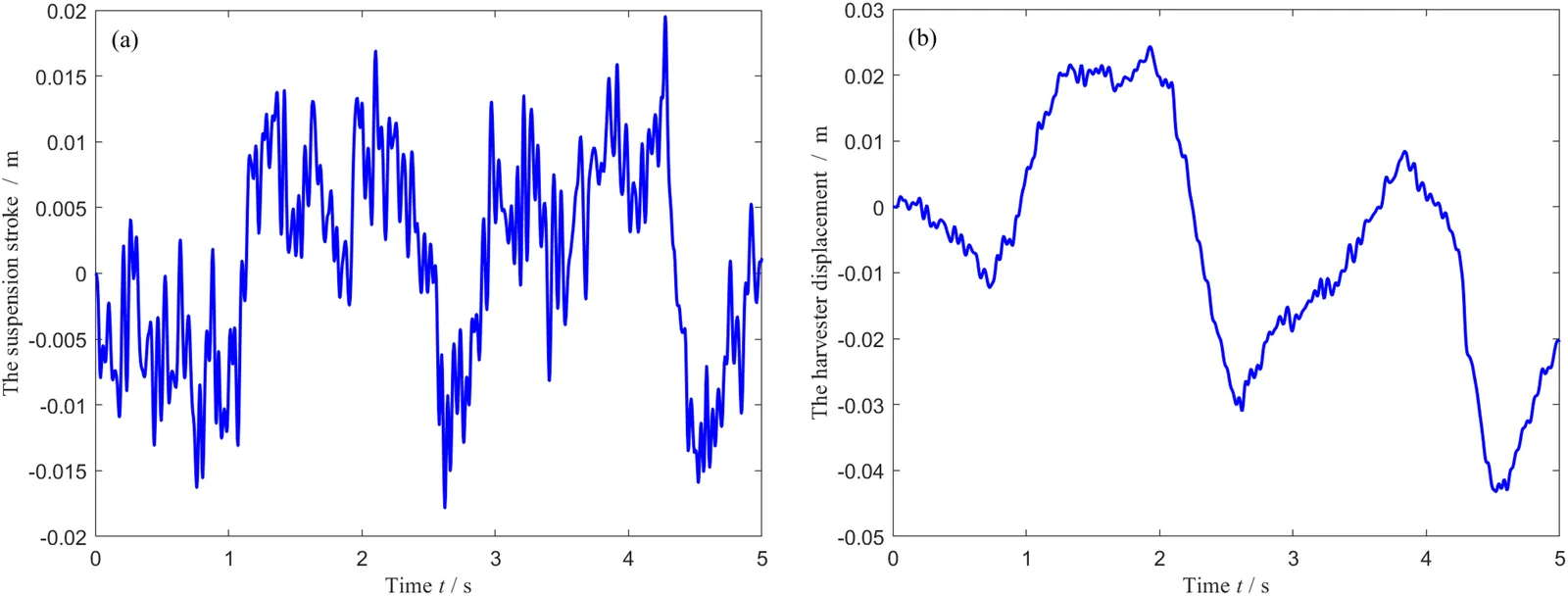
Relative displacements of (a) the suspension and (b) the harvester under the Class C road.

In addition, besides the two optimization objectives, it is also necessary to determine whether the three constraints are satisfied simultaneously. Firstly, the road-handling stability is plotted in Fig 10 and the values are all basically less than 1. Secondly, relative displacements of both the suspension and the harvester are shown in Fig 11. It can be seen that the former is all less than 0.02m and the latter is all less than 0.05m. Therefore, the above results once again verify that the proposed mixed H2/H∞ control method is effective.

### Effects of key parameter

**Fig 12:**
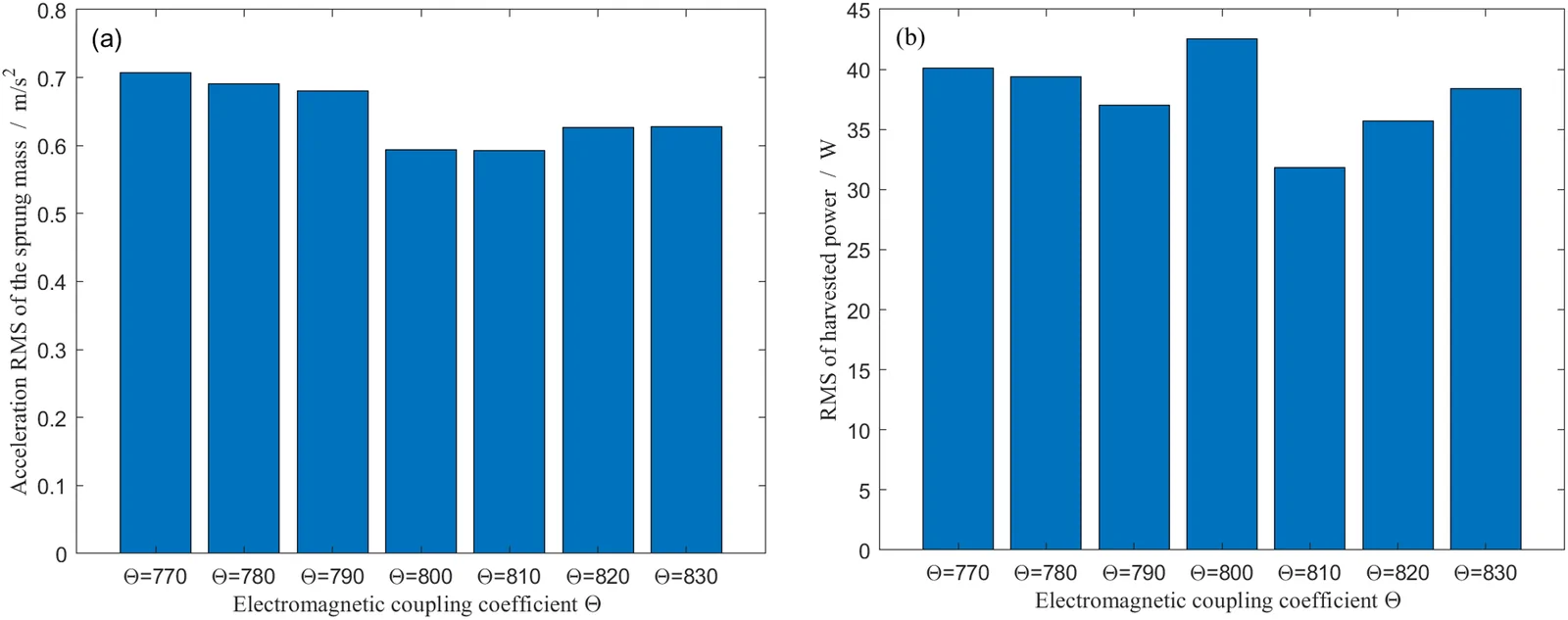
RMSs of the (a) vertical acceleration and (b) harvested power under using different Θ.

Key parameters have a significant impact on the system performance. As for the quarter-suspension SVSEH system, the magnetoelectric coupling coefficient (Θ) is a critical parameter and it serves as an important bridge between vibration suppression and energy harvesting. On one hand, Θ directly determines the amount of the electromagnetic shunt damping force, thereby influencing the vibration suppression performance. On the other hand, Θ directly governs the output voltage of the electromagnetic energy harvester, thereby affecting the vibration energy harvesting performance. To analyze the effects of Θ, RMS values of the vertical acceleration and harvested power are simulated under using different Θ and the results are shown in Fig 12.

It can be observed that: 1) RMS values of both the vertical acceleration and the harvested power fluctuate slightly with Θ, which indicates that the mixed H2/H∞ control strategy is a little robust; 2) In most cases, the greater the RMS value of the vertical acceleration, the greater the RMS value of the harvested energy. This indicates that it is not for arbitrary Θ that the proposed method can reduce the conflict between vibration suppression and energy harvesting; 3) There exists an optimal Θvalue, approximately 800, that enables simultaneously achieving the minimum vertical acceleration and the maximum harvested energy. In a sense, the decoupling between vibration suppression and energy harvesting can be truly realized once Θ is optimized. Furthermore, Θis strongly related to the configuration of the Halbach array and the coils in the electromagnetic energy harvester. As a result, this is worthy of studying their structural optimization, but beyond the scope of this paper.

## Conclusions

Single-objective control methods cannot deal with the conflict between vibration suppression and energy harvesting in the suspension SVSEH system, so that the energy harvesting performance is limited. This paper proposed a bi-objective control method for the quarter-suspension SVSEH system. Main conclusions of this paper may include:

1) The 3-DOF magneto-electromechanical coupling model of the quarter-suspension SVSEH system was built. In order to release the nonlinearity, linearization was applied to deal with the nonlinear restoring force of QZS.
2) The 7-variable state equation of the quarter-suspension SVSEH system was built and vibration suppression and energy harvesting were defined as two independent optimization criteria, respectively. Then the reduced-tradeoff control of the quarter-suspension SVSEH system was equivalent to a bi-objective H2-norm optimization subject to an H∞-norm constraint.
3) The mixed H2/H∞ state feedback-based control strategy was proposed to address the above bi-objective optimization. In particular, a low-pass filter was proposed to ensure the “strictly proper” condition of H2 control. Then LMIs were adopted to solve the mixed H2/H∞ controller.
4) The Simulink model was built to testify the proposed method and the results indicated that the mixed H2/H∞ control strategy performed better than the MIPC strategy. In particular, the tradeoff between vibration suppression and energy harvesting was greatly reduced when Θ was optimized.

While this study provides a novel reduced-tradeoff control framework for SVSEH in vehicle suspensions, it should be acknowledged that there are some limitations. First, the main drawback of this control strategy is its lack of adaptability to varying operating modes. Adaptive control strategies will be an important direction of future research. Secondly, the configuration of the Halbach array and the coil in the electromagnetic energy harvester is not discussed and an optimal magnetoelectric coupling coefficient deserved to be studied for the proposed control strategy. Thirdly, the proposed method was validated only by simulations. Future work will focus on real-world testing.

**Table:** 

Nomenclature			
$c_{s}$	Mechanical damping ($\text{N}\cdot \text{s}\cdot {\text{m}}^{-\text{1}}$)	$F_{a}$	The reactive force (N)
$F_{h}$	The restoring force of the dual-plane spring (N)	$F_{non}$	Nonlinear perturbation term (N)
$F_{s}$	The restoring force of the QZS spring (N)	f	The temporal frequency (Hz)
$f_{\text{0}}$	The temporal cutoff frequency (Hz)	$n_{c}$	The spatial cutoff frequency (rad/m)
g	The gravitational acceleration (m/s^2^)	$k_{h}$	Stiffness of the dual-plane spring ($\text{N}\cdot {\text{m}}^{\text{-1}}$)
i	The induced current (A)	$k_{s}$	Stiffness of the QZS ($\text{N}\cdot {\text{m}}^{\text{-1}}$)
$k_{s\text{1}}$	Linear part of the QZS ($\text{N}\cdot {\text{m}}^{\text{-1}}$)	$k_{u}$	The tire stiffness ($\text{N}\cdot {\text{m}}^{\text{-1}}$)
$L_{coil}$	Total inductance of the coil (mH)	$m_{h}$	The harvester mass (Kg)
$m_{s}$	The sprung mass (Kg)	$m_{u}$	The unsprung mass (Kg)
n	The spatial frequency (rad/m)	$P_{h}$	The harvested power (W)
$R_{coil}$	The resistance of the coil (Ω)	$R_{L}$	The resistive load (Ω)
v	The vehicle velocity (km/h)	$z_{hmax}$	The maximum harvester displacement (m)
$z_{max}$	The suspension stroke (m)	$z_{r}$	The road displacement excitation (m)
$z_{s}$	The displacement of the sprung mass (m)	$z_{u}$	The displacement of the unsprung mass (m)
α	The first-order low-pass filter	Θ	The magnetoelectric coupling coefficient
$\omega_{\text{0}}$	The angular frequency (rad)	γ	The performance metric

## Supporting information

S1 Datasets Measured acceleration, displacement and harvested power under Class C road excitation. (RAR)
